# Oxidative Stress and Ischemia/Reperfusion Injury in Kidney Transplantation: Focus on Ferroptosis, Mitophagy and New Antioxidants

**DOI:** 10.3390/antiox11040769

**Published:** 2022-04-12

**Authors:** Simona Granata, Valentina Votrico, Federica Spadaccino, Valeria Catalano, Giuseppe Stefano Netti, Elena Ranieri, Giovanni Stallone, Gianluigi Zaza

**Affiliations:** 1 Renal Unit, Department of Medicine, University Hospital of Verona, 37124 Verona, Italy; simona.granata@unifg.it (S.G.); valentina.votrico@studenti.univr.it (V.V.); 2 Nephrology, Dialysis and Transplantation Unit, Department of Medical and Surgical Sciences, University of Foggia, 71122 Foggia, Italy; giovanni.stallone@unifg.it; 3 Clinical Pathology, Center of Molecular Medicine, Department of Medical and Surgical Sciences, University of Foggia, 71122 Foggia, Italy; federica.spadaccino@unifg.it (F.S.); valeria.catalano@unifg.it (V.C.); giuseppestefano.netti@unifg.it (G.S.N.); elena.ranieri@unifg.it (E.R.)

**Keywords:** ischemia/reperfusion injury, oxidative stress, ferroptosis, mitophagy, kidney transplantation

## Abstract

Although there has been technical and pharmacological progress in kidney transplant medicine, some patients may experience acute post-transplant complications. Among the mechanisms involved in these conditions, ischemia/reperfusion (I/R) injury may have a primary pathophysiological role since it is one of the leading causes of delayed graft function (DGF), a slow recovery of the renal function with the need for dialysis (generally during the first week after transplantation). DGF has a significant social and economic impact as it is associated with prolonged hospitalization and the development of severe complications (including acute rejection). During I/R injury, oxidative stress plays a major role activating several pathways including ferroptosis, an iron-driven cell death characterized by iron accumulation and excessive lipid peroxidation, and mitophagy, a selective degradation of damaged mitochondria by autophagy. Ferroptosis may contribute to the renal damage, while mitophagy can have a protective role by reducing the release of reactive oxygen species from dysfunctional mitochondria. Deep comprehension of both pathways may offer the possibility of identifying new early diagnostic noninvasive biomarkers of DGF and introducing new clinically employable pharmacological strategies. In this review we summarize all relevant knowledge in this field and discuss current antioxidant pharmacological strategies that could represent, in the next future, potential treatments for I/R injury.

## 1. Introduction

Kidney transplantation represents the most cost-effective modality of renal replacement therapy for patients with irreversible chronic kidney failure (end-stage renal disease, stage 5 chronic kidney disease) [1]. However, despite continuous technical and pharmaceutical progress in transplant medicine, some patients develop early acute post-transplant complications and experience a slow recovery of the renal function with the need for dialysis (generally during the first week after transplantation). This clinical condition, namely delayed graft function (DGF), has a significant social and economic impact as it is associated with prolonged hospitalization [2], poly-pharmacological approaches (particularly in the presence of concomitant acute allograft rejection) [3], and shorter graft survival [4].

The risk of DGF is higher in specific organ transplant programs using kidneys from non-heart-beating, elderly, multimorbid (e.g., diabetes, hypertension) donors, recipients with a previous allograft failure and/or allosensitized, and organs damaged by acute kidney injury and prolonged cold ischemia time [5,6].

Particularly, during ischemia, the significant reduction in oxygen supply and the consequent cellular switch from an aerobic to an anaerobic metabolism, may decrease the rate of ATP production [7] and cause the accumulation of lactate (leading to acidosis). Consequently, Na+/K+ ATPases, Na+/H+ and Ca2+-ATPase pumps can become dysfunctional, and sodium, hydrogen and calcium accumulate in the cytoplasm with consequent hyper-osmolality, a rise in water transport across cell membranes, and cellular swelling [8].

During reperfusion, the rapid increase of oxygen and pH normalization, may enhance cytosolic calcium concentration activating cysteine proteases (e.g., calpains, caspases) and triggering the apoptotic pathway. Moreover, calcium overload stimulates the opening of the mitochondrial permeability transition pores (mPTP) that allow the release of substances such as cytochrome C, succinate and mitochondrial DNA which can induce cell death through apoptosis and necrosis and act as danger/damage-associated molecular patterns (DAMP) promoting activation of both the innate and adaptive immunity [9,10,11]. In addition, these mechanisms may result in progressive interstitial fibrosis [12,13,14].

Furthermore, the overproduction of reactive oxygen species (ROS) following ischemia/reperfusion (I/R) may be induced by deregulation of numerous enzymes able to reduce molecular oxygen forming superoxide and/or hydrogen peroxide such as NADPH oxidase, nitric oxide synthase (NOS), the mitochondrial electron transport chain, and xanthine oxidoreductase (XOR).

XOR is a complex molybdoflavoenzyme that controls the rate-limiting step of purine catabolism and exists in two interconvertible forms, xanthine dehydrogenase (XDH) and xanthine oxidase (XO). XDH preferably uses NAD+ as an electron acceptor, while XO uses O_2_ as the terminal electron acceptor thereby exhibiting the ability to generate ROS [15]. This enzyme converts hypoxanthine into xanthine generating superoxide (O_2_^−^) and hydrogen peroxide (H_2_O_2_) that play an important role in mediating the recruitment and/or activation of leukocytes that orchestrate the tissue damage [16].

Additionally NADPH oxidases, multimeric complexes that generate superoxide or H_2_O_2_, composed of seven family members (NOX1–5, DUOX1–2) [17], are implicated in the production of ROS following I/R. NOX enzymes use oxygen as final electron acceptors via NADPH, FAD, and heme groups. The DUOX enzymes predominately produce hydrogen peroxide along with NOX-4, while the remaining NOX isoenzymes largely produce superoxide. NOXs are constitutively inactive and require cell stimulation to translocate to the membrane and generate ROS [16]. In I/R this enzymatic complex may be activated by several chemical mediators that are produced and released by cells, such as: hypoxia inhibitory factor-1α (HIF-1α) [18], phospholipase A2 [19], arachidonic acid [20], complement system [21], cytokines such as TNF-α and IL-1β from macrophages and mast cells [22].

Another source of ROS is the uncoupled NOS that produces nitric oxide (NO) by the conversion of L-arginine to L-citrulline using NADPH as a reducing substrate and tetrahydrobiopterin (BH4) as a redox-sensitive cofactor. This enzyme, under hypoxic conditions may be converted to an O_2_-generating enzyme due to the reduced concentration of BH4, increasing the oxidative damage [23].

Furthermore, mitochondria, organelles that generate most of the chemical energy needed to power the cell, contribute to ROS production through univalent reduction of O_2_ mainly by the leakage of electrons at complex I and α-ketoglutarate dehydrogenase [24].

During ischemia, the alteration of mitochondrial structure, the high NADH/NAD+ ratio, and the accumulation of the citric acid cycle metabolite succinate exacerbate this process [25,26].

Oxidative stress, then, plays a key role in organ damage after I/R by activating ferroptosis, an iron-driven cell death characterized by iron accumulation, excessive ROS and lipid peroxidation products and mitophagy, the selective degradation of damaged mitochondria by autophagy.

## 2. Ferroptosis: Role in Kidney Allograft I/R Injury

Ferroptosis is a form of regulated cell death driven by iron accumulation, lipid peroxidation and subsequent plasma membrane rupture [27]. It is mainly characterized by: a nucleus lacking chromatin condensation, mitochondria with reduced volume and cristae, significant cell enlargement and plasma membrane rupture [28,29].

**Figure 1:**
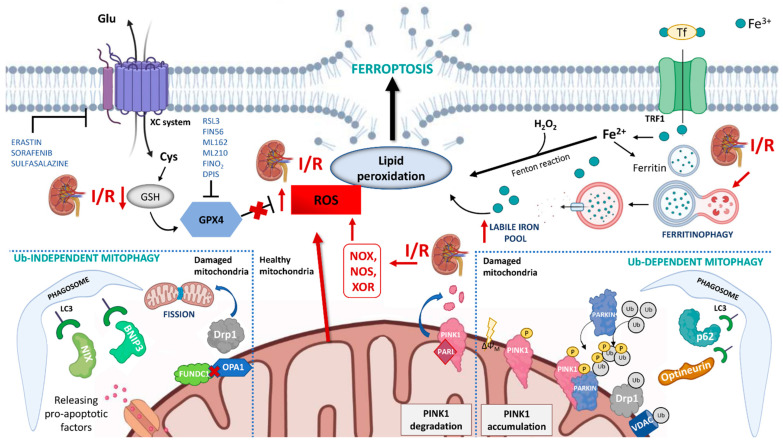
Schematic representation of the mechanisms of ferroptosis and mitophagy in renal ischemia/reperfusion (I/R) injury. During I/R several pathways contribute to ferroptosis: (i) the overproduction of ROS by NADPH oxidase (NOX), nitric oxide synthase (NOS), xanthine oxidoreductase (XOR) and mitochondria promotes lipid peroxidation and plasmatic membrane rupture; (ii) the reduction in glutathione (GSH) content inhibits glutathione peroxidase 4 (GPX4) activity and its protective action against membrane lipid peroxidation; (iii) I/R can indirectly induce ferritinophagy which causes the degradation of intracellular ferritin, and the increment of intracellular labile iron pool. Mitophagy is activated in I/R through both ubiquitin-dependent and ubiquitin-independent mechanisms and seems to have a protective role in I/R injury by reducing the release of reactive oxygen species from dysfunctional mitochondria. In physiological conditions, PINK1 is imported into mitochondria where it is cleaved by the intramembrane serine protease presenilin associated rhomboid-like (PARL) and ultimately degraded. When mitochondria are damaged, and lose their membrane potential, PINK1 accumulates on the mitochondrial outer membrane (MOM) and recruits Parkin. Parkin ubiquitinates several mitochondrial substrates such as voltage-dependent anion-selective channel protein (VDAC) and dynamin-1-like protein (DRP1). These ubiquitinated proteins can recruit mitophagy receptors (such as optineurin, p62) that link mitochondria to autophagosomes through interacting with LC3. This causes an autophagic engulfment of the organelle necessary for its degradation. The ubiquitin-independent mechanism is regulated by mitophagy receptors that localize on MOM, such as BCL2 interacting protein 3 (BNIP3), BNIP3-like (BNIP3L/NIX), and FUN14 domain containing 1 (FUNDC1). These proteins bridge mitochondria to autophagosome by directly interacting with LC3.

In the context of renal I/R, the iron accumulation, through the Fenton reaction, may generate a large amount of ROS (also increased by the concomitant mitochondrial dysfunction and NOX family activity) that can severely enhance intra-cellular oxidative stress and lipid peroxidation (Figure 1).

Two pathways may trigger ferroptosis: the extrinsic and the intrinsic pathway [27]. The extrinsic pathway is initiated through the inhibition of the cystine/glutamate exchanger of the membrane, namely the XC system, that mediates the entry of cystine into the cells, which is used to synthesize glutathione (GSH) [30], a cofactor used by glutathione peroxidase 4 (GPX4) to eliminate lipid peroxides in the cell membranes. Therefore, inhibition of the XC system indirectly reduces the activity of GPX4 with consequent accumulation of lethal lipid peroxides and induction of ferroptosis. Several agents such as erastin, sulfasalazine, and sorafenib, by blocking the XC system, are able to elicit ferroptosis through this mechanism.

The intrinsic pathway is mainly induced by drugs or small-molecule inhibitors such as RSL3, ML162, ML210, FIN56 and FINO2 which can directly or indirectly inhibit GPX4 activity [31]. Additionally, the molecules regulating iron uptake, storage, and utilization (such as ferritin, transferrin, and lactotransferrin) can influence ferroptosis by increasing levels of labile iron (free-iron source that was relatively accessible for Fenton reaction) in the cell [32]. Transferrin and lactotransferrin are proteins responsible for iron transport that, binding to their receptors, mediate the import of Fe into the cytoplasm. Ferritin is the intracellular iron-storage protein that can be degraded by lysosomes in a process termed ferritinophagy and increases free iron levels thus promoting ferroptosis [33] (Figure 1).

Contrarily enzymatic and non-enzymatic systems (CoQ10, vitamin E, ferrostatins, and liproxstatins), together with membrane repair systems, prevent lipid peroxidation and protect the cells from ferroptosis [34,35,36,37].

Recent studies have reported that ferroptosis may be involved in the pathophysiological pathway associated with the I/R injury [29,38].

Su et al. [39] demonstrated that pannexin 1, a membrane channel involved in regulating ATP release as a DAMP molecule able to activate apoptosis or autophagy signaling in oxidative condition [40,41], may activate ferroptosis in a mouse model of renal I/R injury [39]. Knockout of the panx1 gene in mice subjected to I/R is associated with a lower increment of serum creatinine and decreased tubular cell death together with decreased lipid peroxidation compared with wild-type mice. This protective effect seemed mediated by the inactivation of the MAPK/ERK pathway and the up-regulation of the antioxidant gene heme oxygenase-1 (HO-1).

The anti- ferroptosis protective effects may also be exerted by the activity of the Augmenter of Liver Regeneration (ALR), a sulfhydryl oxidase enzyme localized in the intermembrane space of mitochondria. This enzyme participates in the “disulfide relay system” that mediates the import of proteins to the intermembrane space [42] and has anti-apoptotic and anti-oxidative properties. ALR expression was significantly increased in ischemic rats and the administration of recombinant human ALR, by enhancing the proliferation of renal tubular cells and attenuating tubular cell apoptosis, effectively reduced tubular injury and ameliorated the impairment of renal function [43,44].

The protective role of ALR in ferroptosis could also be mediated by a reduction of ROS levels via its interaction with the GSH-GPX4 system [45] and by promoting the clearance of damaged mitochondria (a mechanism called mitophagy) [46].

Therefore, ALR activation may represent a possible future prevention therapeutic strategy for I/R-induced allograft injury.

## 3. Mitophagy: Another Player in Kidney Allograft I/R Injury

Damaged or dysfunctional mitochondria harm the cell by producing a large amount of ROS and releasing pro-apoptotic factors. Thus, timely removal of these organelles is critical to cellular homeostasis and viability [47].

Mitophagy is the mechanism of selective degradation of damaged mitochondria via autophagy [48] that is executed by a ubiquitin-dependent and ubiquitin-independent pathway. The former is regulated by the PTEN-induced putative kinase 1 (PINK1)-Parkin pathway. PINK1 is a mitochondrial serine/threonine kinase and Parkin is a cytosolic ubiquitin E3 ligase. In physiological conditions, PINK1 is imported into mitochondria where it is cleaved by the intramembrane serine protease presenilin associated rhomboid-like (PARL) and ultimately degraded [49]. When mitochondria are damaged and lose their membrane potential, the import of PINK1 is hindered leading to an accumulation of this kinase at the mitochondrial outer membrane (MOM). Subsequently, PINK1 recruits Parkin and activates its ligase activity [50]. Parkin ubiquitinates several mitochondrial substrates such as Mitofusin 2 (Mfn2), voltage-dependent anion-selective channel protein (VDAC), and dynamin-1-like protein (DRP1). These ubiquitinated proteins can recruit mitophagy receptors (such as optineurin, p62, NBR1) that link mitochondria to autophagosomes through interacting with LC3. This causes an autophagic engulfment of the organelle necessary for its degradation [49,51].

The ubiquitin-independent mechanism is regulated by mitophagy receptors that localize on MOM such as BCL2 interacting protein 3 (BNIP3), BNIP3-like (BNIP3L/NIX), and FUN14 domain containing 1 (FUNDC1) [52,53]. These proteins bridge mitochondria to autophagosome by directly interacting with LC3 [54] (Figure 1).

Mitophagy is also regulated by proteins that participate in mechanisms of fusion and fission of these organelles. Fusion results in a single mitochondrion being formed from previously independent structures [55], generating networks with continuous membranes and matrix lumen [56]. Fission produces one or more daughter organelles and, in the case of reduced mitochondrial membrane potential, segregates this organelle for elimination by autophagy [56].

The coordination of fission/fusion and mitophagy seems to be mediated by FUNDC1. In physiological conditions, this receptor anchors dynamin-related GTPases optic atrophy 1 (OPA1) toward the inner surface of the MOM. In response to mitochondrial stress, the disassembly of the FUNDC1–OPA1 complex and the recruitment of Drp1 promote mitochondrial fission and mitophagy [57].

This complex and fascinating multifactorial autophagic mechanism may play a protective role in allografts undergoing I/R injury.

Deficiency of BNIP3 or Pink1 and/or Parkin in rat models of renal I/R injury resulted in increment of ROS production, apoptosis, and tubulointerstitial inflammation [58,59,60,61]. The same effects were obtained by the suppression of the mitophagic cascade by acting on proteins regulating fission (e.g., Drp1) or fusion (e.g., OPA1) [62,63].

The protective effects of mitophagy on kidney undergoing I/R injury were observed after ischemic preconditioning [64], a short period of non-lethal ischemia-reperfusion that protect solid organ against subsequent extended I/R injury [65]. The up-regulation of mitophagy via the PINK1-Parkin pathway improved mitochondrial function, minimized ROS production and enhanced cell survival [64].

All these findings suggest that mitophagy, preserving mitochondrial quality and tubular cell survival, could represent a valuable protective mechanism against I/R injury that should be promoted by pharmacological interventions.

## 4. Antioxidants and Ferroptosis/Mitophagy Regulators

Several pharmacological agents with anti-oxidant potentials have been proposed for the treatment of I/R injury, including those targeting the nuclear factor erythroid 2–related factor 2 (Nrf2), hydrogen sulfide (H_2_S), mitochondria-targeting antioxidants, drugs with anti-oxidant potential, and other specific ferroptosis and mitophagy regulators (Table 1).

### 4.1. Regulation of the Nuclear Factor Erythroid 2–Related Factor 2 (Nrf2)

**Figure 2:**
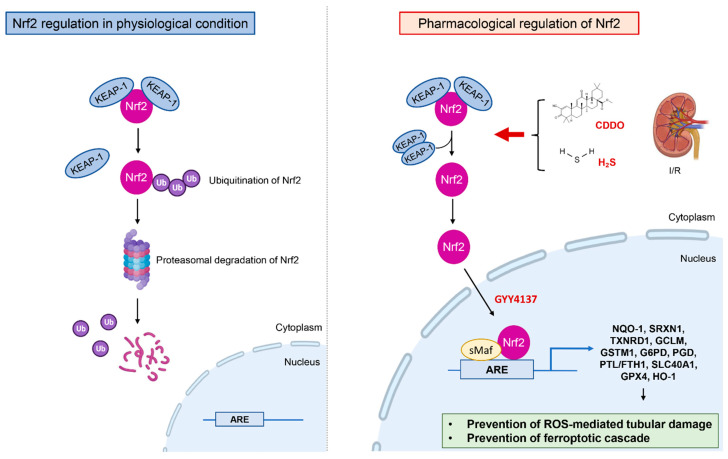
Mechanism of Nrf2 regulation in the treatment of renal I/R. In physiological condition Nrf2 binds to Kelch-like ECH-associated protein-1 (Keap1) in the cytoplasm and is degraded by ubiquitin-proteasome pathway. During renal I/R the hyperactivation of Nrf2 by CDDO, H_2_S, water-soluble H_2_S donor (such as GYY4137) leads to nuclear traslocation of Nrf2 that binds to antioxidant response elements and activates transcription of the genes encoding proteins involved in antioxidants mechanisms and iron metabolism thereby preventing the ROS-mediated tubular damage and the ferroptotic cascade.

The nuclear factor erythroid 2–related factor 2 (Nrf2) is an inducible transcription factor that regulates the expression of antioxidant response elements [66] (Figure 2).

In physiological conditions Nrf2 binds to Kelch-like ECH-associated protein-1 (Keap1) in the cytoplasm and is degraded by the ubiquitin-proteasome pathway [67]. Under oxidative stress, Nrf2 escapes from degradation thanks to the inactivation of Keap1, forms dimers with a member of the small Maf proteins in nuclei, binds to anti-oxidant response elements, and activates transcription of the antioxidant genes [68].

In the course of renal I/R, the hyperactivation of Nrf2 by 1-[2-cyano-3-,12-dioxooleana-1,9(11)-dien-28-oyl] imidazolide (CDDO) in the initial phase of the ischemia process prevents the progression of ROS-mediated tubular damage by inducing the expression of genes involved in anti-oxidant response [NADPH: quinone acceptor oxidoreductase 1 (Nqo1), Sulfiredoxin-1 (Srxn1) and Thioredoxin Reductase 1 (Txnrd1)], glutathione metabolism [Glutamate-Cysteine Ligase Modifier Subunit (Gclm) and Glutathione S-Transferase Mu 1 (Gstm1)], and NADPH synthesis [Glucose-6-Phosphate Dehydrogenase (G6pd) and Phosphogluconate Dehydrogenase (Pgd)] [69].

Nrf2 also regulates the expression of genes encoding for proteins mediating iron metabolism and is able to prevent the ferroptotic cascade, such as ferritin light and heavy chain (FTL/FTH1), ferroportin (SLC40A1) [70,71], GPX4, and HO-1, by which ferroptosis is inhibited and I/R-associated kidney injury alleviated [72,73].

Contrarily, silencing Nrf2 in mice undergoing I/R injury, triggered worse renal function and elevated histological tubular damage, increased renal vascular permeability, oxidative stress, and apoptosis compared to wild-type mice [74,75,76].

### 4.2. Antioxidant Effects of Hydrogen Sulfide (H_2_S)

Hydrogen sulfide (H_2_S) is a membrane-permeable, gaseous mediator that inhibits oxidative damage through scavenging free radicals and ROS by increasing the level of GSH and thioredoxin, and the activation of Nrf2 signaling by inactivation of Keap1 [77,78].

Several studies have reported the protective effect of soluble forms of H_2_S (such as sodium hydrosulfide or sodium sulfide) in animal models of I/R injury [79,80,81,82,83,84] (Table 2).

During renal I/R injury, the expression of the enzyme cystathionine gamma-lyase that catalyzes H_2_S formation is up-regulated and consequently, H_2_S production, as well as its plasmatic concentration, increased [80]. This could represent a defensive mechanism of the kidney against I/R. In fact, the administration of exogenous NaHS (15 min before ischemia and 5 min before reperfusion) prevented the I/R-induced activation of caspase-3 as well as the decline in the expression of the apoptotic markers Bid and Bcl-2 [79] with positive functional and histological effects.

Another protective mechanism mediated by H_2_S is based on its ability to induce hypometabolism (50% reduction in oxygen consumption and 60% in carbon dioxide output) [85]. The demand for O_2_ is reduced to such an extent that H_2_S-treated mice can survive in 5% O_2_ for over 6 h [86].

In a mouse model of renal I/R injury, H_2_S administrated before the ischemic insult may preserve renal function, prevent apoptosis and limit the influx of leukocytes and granulocytes into the renal interstitium [82]. Contrarily, a post-ischemic treatment with H_2_S may not exert any protective effects. These results demonstrated that the reduction in O_2_ demand during hypoxia prevents the activation of detrimental pathways associated with I/R [82].

According to these findings, Han et al. demonstrated, in an ischemic kidney mouse model, the capability of NaHS treatment to accelerate the regeneration of damaged tubular cells by activating anti-oxidant effects [83].

More recently Zhao et al. also found that a water-soluble H_2_S donor (GYY4137) was able to attenuate the deterioration of renal function and morphology in the renal I/R model by increasing the nuclear localization of Nrf2 [84].

These findings indicate that the H_2_S-producing system may play a critical role in the recovery from acute kidney injury and prevention of progression to chronic kidney disease.

### 4.3. Mitochondria-Targeting Antioxidants

The commonly used antioxidants could be ineffective in limiting mitochondrial ROS production, due to their low penetrance to the mitochondria interior. To overcome these limitations, mitochondria-targeting anti-oxidants have been developed to provide their delivery to the mitochondrion interior [87]. These molecules have been used in numerous pre-clinical and clinical studies (Table 3) [88,89,90,91,92,93,94,95,96,97,98,99,100,101,102].

MitoQ: a quinone comprises a lipophilic triphenylphosphonium (TPP) cation covalently linked by an aliphatic 10-carbon chain to an antioxidant ubiquinone moiety [103]. The TPP lipophilic cation passes rapidly through biological membranes and its positive charge drives the extensive accumulation of these molecules into mitochondria where it acts as a chain-breaking anti-oxidant to prevent oxidative damage [104].

In a mouse model of bilateral renal ischemia, followed by up to 24 h reperfusion, intra-venous administration of MitoQ 15 min prior to ischemia reduced the severity of I/R injury to the kidney by decreasing oxidative damage [88,89].

Its ability to preserve mitochondrial integrity and function limits ferroptosis induced by loss of GPX4 or exposure to RSL3 [105].

Szeto-Schiller peptide SS-31 (also known as MTP-131, elamipretide, and bendavia) is a peptide agent that interacts with cardiolipin [106] in the inner mitochondrial membrane and exerts strong anti-oxidant propriety [107].

In a rat model of renal I/R injury, treatment with SS-31 protected mitochondrial structure and respiration during early reperfusion, accelerated recovery of ATP, reduced apoptosis and necrosis of tubular cells, and abrogated tubular dysfunction [93]. In addition, SS-31 seemed to be able to modulate the expression of members of the RAS system (an important regulator of kidney functions), in particular aminopeptidase A (APA) and Ang receptors (AT2R) [94].

In a recent Phase 2a prospective, multicenter, randomized, double-blind, placebo-controlled study Saad et al., assessed the safety, tolerability, and efficacy of IV administered elamipretide (clinical formulation of SS-31) for reduction of reperfusion injury in patients with severe atherosclerotic renal artery stenosis undergoing revascularization with percutaneous transluminal renal angioplasty (PTRA) [95]. Patients were treated before and during PTRA with elamipretide (0.05 mg/kg per hour intravenous infusion) or placebo. Compared to the placebo group, the patients who received elamipretide showed increased estimated GFR and a decline in systolic blood pressure after 3 months.

Tempol (4-hydroxy-2,2,6,6-tetramethylpiperidine-N-oxyl or 4-hydroxy-tempo) is a stable piperidine nitroxide that scavenges superoxide anions and reduces the intracellular concentrations of Fe2+ and, hence, the formation of hydroxyl radicals via the Fenton or Haber-Weiss reactions [108,109].

In a rat model of renal I/R injury, administration of tempol prior to and throughout reperfusion attenuated renal dysfunction at least partially through reduced renal activity of myeloperoxidase (MPO) and levels of malondialdehyde (MDA) [100].

This compound is currently under investigation in a clinical trial evaluating its ability to prevent many of the toxicities associated with cisplatin and radiation treatment (including the prevention of mucositis, nephrotoxicity, and ototoxicity) in head and neck cancer patients (NCT03480971).

Mito-TEMPO is a combination of the intracellular anti-oxidant piperidine nitroxide TEMPO (2,2,6,6-tetramethylpiperidin-1-yloxy) and the TPP cation which facilitates 1000-fold accumulation into the mitochondrial matrix and selectively targets mitochondrial ROS [110]. Administration of mito-TEMPO in rats after reperfusion and for 3 or 5 consecutive days after surgery restored the renal mtDNA level, mitochondrial mass, and ATP production with a consequently reduced inflammation and kidney injury [101].

XJB peptides are composed of 4-NH2-TEMPO, a stable nitroxide radical with anti-oxidant properties conjugated to a pentapeptide fragment from gramicidin S (Leu-d-Phe-ProVal-Orn), a natural membrane-active cyclopeptide antibiotic localized in the inner mitochondrial membrane [111]. The most studied of all the XJB peptides is XJB-5-131. Mice injected intraperitoneally with XJB-5-131 (10 mg/kg) 30 min prior to ischemia and for 3 consecutive days after surgery showed decreased kidney inflammation, regeneration and repair of injured renal tubular cells at least partially through the inhibition of I/R induced ferroptosis [102].

### 4.4. Drugs with Antioxidant Properties

Dexmedetomidine is a highly selective and specific α2-adrenoreceptor agonist with a sedative effect.

In a rat model of I/R, dexmedetomidine, administered intraperitoneally at different dosages (from 10 to 100 ug/kg) at the starting of ischemia or reperfusion or after surgery, attenuated renal dysfunction, acute tubular necrosis and inflammatory response at least partially through increased renal p38 MAPK, anti-oxidant levels, and maintenance of autophagy [112,113,114,115].

Edaravone (3-methyl-1-phenyl-2-pyrazolin-5-one) is a potent scavenger of hydroxyl and peroxyl radicals. As recently reported in the literature, administration of edaravone (from 3 to 10 mg/kg) intravenously in a mouse model of I/R injury (by clamping of renal arteria) protected against kidney damage by reducing oxidative stress, inhibiting apoptosis, and improving mitochondrial injury through JAK/STAT signaling [116,117].

In the future, edaravone could be potentially employable in clinical organ preservation and transplantation.

### 4.5. Ferroptosis and Mitophagy Specific Agents:

Besides the aforementioned antioxidant agents that can have an indirect role on both ferroptosis and mitophagy, specific molecules have been proposed for the direct regulation of these two pathways, including ferrostatin-1 and liproxstatin, two specific inhibitors of ferroptosis that because of their reactivity as radical trapping antioxidants may allow to reduce the accumulation of lipid hydroperoxides [118]. Liproxstatin-1 was reported to be able to suppress ferroptosis in human renal proximal tubule epithelial cells, in Gpx4−/− kidney, and in an I/R-induced tissue injury models [37]. However, additional studies (including clinical trials) should be undertaken to better address the clinical utility of these agents.

## 5. Conclusions

There are no therapeutic strategies available in clinical practice to slow down the onset and development of the allograft damage induced by I/R injury. However, data obtained in vitro and in animal models suggest that modulation of ferroptosis and mitophagy could represent a future therapeutic tool to prevent or slow-down the progression of the allograft I/R injury. Moreover, some of the components of both biological mechanisms could be proposed as novel (and not invasive) early diagnostic biomarkers for I/R injury-induced allograft complications (mainly delayed graft function).

## Figures and Tables

**Table 1:** Antioxidant molecules with their class, mechanism and targets.

Molecule	Class	Mechanism and Targets
Nrf2	Transcription factor	In response to oxidative stress, Nrf2 escapes from degradation throught the inactivation of Keap1 and binds to antioxidant rensponse elements in the regulatory region of target genes. Nrf2 induces the expression of genes encoding proteins involved in redox homeostasis, xenobiotic metabolism, anabolic metabolism, DNA damage, proliferation and survival responses
H_2_S	Gaseous mediator	H_2_S exerts anti-oxidant effects through several mechanisms: (i) acts as a direct scavenger that reduces excessive amounts of ROS; (ii) upregulates the antioxidant defense system through the Nrf2 pathway; (iii) increases the production of intracellular GSH
Dexmedetomidine	Drug (a2-adrenoreceptor agonist with sedative effect)	Dexmedetomidine increases antioxidant activity and reduces the synthesis of ROS, but the exact mechanism has not yet been fully elucidated
Edaravone	Neuroprotective drug	Edaravone is a scavenger of hydroxyl and peroxyl radicals
Ferrostatin-1	Arylamine	Radical-trapping anti-oxidants
Liproxstatin	Arylamine	Radical-trapping anti-oxidants
MitoQ	Quinone	MitoQ is accumulated at the matrix-facing surface of the inner mitochondrial membrane, where complex II of the ETC recycles it into the active ubiquinol form (MitoQH2). This form has been shown to be a highly effective anti-oxidant by reacting with ROS
SS-31	Peptide-based cell-permeable antioxidant compound	SS-31 can scavenge H_2_O_2_ and ONOO^−^ and inhibit lipid peroxidation
Tempol	Superoxide dismutase-mimetic	Tempol scavenges H_2_O_2_, NO, ONOO^−^, lipid peroxyl, and alkoxyl radicals
Mito-TEMPO	Piperidine nitroxide TEMPO combined with the TPP cation	Mito-TEMPO possesses O_2_^−^ and alkyl radical scavenging properties
XJB-5-131	4-NH2-TEMPO combined with pentapeptide fragment from gramicidin S	XJB-5-131 is both an electron scavenger and an anti-oxidant

**Table 2:** Studies reporting the beneficial effects of H_2_S in animal models of I/R injury.

Model	Treatment	Effects	Ref
Ischemic rats	NaHS (100 umol/kg, 2 mL/kg) was administered topically onto the kidneys 15 min before ischemia and 5 min before reperfusion	Reduced renal dysfunction through both anti-apoptotic and anti-inflammatory effects secondary to modulation of the signaling pathways leading to activation of MAPK and NF-kB	[79]
Ischemic mice	NaHS (100 μmol/kg, 8 mL/kg, i.p.) was administered 30 min prior to ischemia and 6 h into reperfusion	Reduced renal dysfunction	[80]
Mouse embryonic fibroblasts	Cells were treated with menadione	H_2_S stabilized Nrf2 through inhibition of Keap1 with consequent Nrf2-mediated induction of cytoprotective genes	[81]
Ischemic mice	H_2_S was administered in 3 different treatment regimens: PRE-TREATMENT (H_2_S 100ppm administered for 30 min before ischemia and last for 25 min during ischemia); POST-TREATMENT (H_2_S 100 ppm administered 5 min before reperfusion); PRE- and POST-TREATMENT (H_2_S 100ppm starting 30 min before ischemia until 30 min after reperfusion)	The H_2_S-induced reduction in metabolism before ischemia (PRE-TREATMENT/PRE- and POST-TREATMENT) protected against acute tubular necrosis, apoptosis, loss of mitochondrial integrity and mitochondrial swelling associated with I/R injury. The protection was less pronounced when H_2_S was administered after the hypoxic period (POST-TREATMENT)	[82]
Ischemic mice	Mice received daily intraperitoneal administration of sodium hydrosulfide hydrate (NaHS; 500 μg/kg) beginning 2 days after ischemia until 8 days after surgery	Exogenous supplement of H_2_S by NaHS after ischemia improved recovery of kidney function by accelerating tubular epithelial cell proliferation, suppressing interstitial cell proliferation and fibrosis. Furthermore, NaHS treatment reduced post-I/R oxidative stress by prevention of reduction of glutathione level	[83]
Ischemic mice	Mice received GYY4137 (H_2_S donor) 50 mg/kg via intraperitoneal injection for 2 consecutive days before ischemia/reperfusion	GYY4137 attenuated the deterioration of renal function and morphology by increasing the expression of anti-oxidant enzymes via activation of the Nrf2 pathway	[84]

**Table 3:** Main published preclinical and clinical studies investigating mitochondria-targeting anti-oxidants.

Molecule	Type of Study	Model/Disease	Treatment	Results	Ref
MitoQ	Preclinical study	Animal model of I/R injury	MitoQ (4 mg/kg) was administered to the mice intravenously 15 min prior to ischemia	MitoQ attenuated renal dysfunction through a reduction in oxidative damage	[88,89]
	Clinical studies	To evaluate the efficacy of MitoQ for improving physiological function (vascular, motor, and cognitive) in middle-aged and older adults (≥60 years)	Oral supplementation of MitoQ (20 mg/day for 6 weeks)	MitoQ improved endothelial function, reduced aortic stiffness and decreased plasma oxidized LDL without altering circulating markers of inflammation or traditional cardiovascular disease risk factor	[90]
		Treatment of patients with Parkinson’s Disease	Two doses of MitoQ (40 or 80 mg once daily) for a period of 12 months versus placebo	MitoQ did not slow the progression of Parkinson’s Disease	[91]
		A Phase 2, randomized, double-blind, parallel design trial to evaluate the ability of MitoQ to reduce raised serum alanine transaminase (ALT) seen in patients with chronic Hepatitis C compared with placebo	Two doses of MitoQ (40 or 80 mg once daily) for 28 days	Both treatment groups showed significant decreases in absolute and percentage changes in serum ALT from baseline to treatment day 28	[92]
SS-31 (Elamipretide, Bendavia, MTP-131)	Preclinical study	Animal model of I/R injury	SS-31 (2.0 mg/kg per day) was administered for 6 weeks, starting 1 month after ischemia	SS-31 restored mitochondria structure in endothelial cells, podocytes, and tubular cells with consequent restoration of peritubular and glomerular capillaries, preservation of podocyte architecture, suppression of inflammation, and fibrosis	[93]
		Mice treated with aristolochic acid or adriamycin to induce acute kidney injury	SS-31 (3 mg/kg) was administered intraperitoneally once a day, starting 1 day before the disease-inducing drugs and then daily until day 6	SS-31 modulated the expression of of members of the RAS system	[94]
	Clinical studies	Patients with severe atherosclerotic renal artery stenosis scheduled for percutaneous transluminal renal angioplasty (PTRA)	Patients were treated before and during PTRA with elamipretide (0.05 mg/kg per hour intravenous infusion) or placebo	Adjunctive elamipretide during PTRA was associated with attenuated postprocedural hypoxia, increased renal blood flow, and improved kidney function	[95]
		Phase 2a, randomized, double-blind, placebo-controlled trial enrolling 300 patients with a first-time anterior STEMI and an occluded proximal or mid-left anterior descending artery undergoing primary percutaneous coronary intervention (PCI) that evaluated the efficacy and safety of Bendavia	Patients were randomized to receive either Bendavia at 0.05 mg/kg per hour or a placebo	Treatment with MTP-131 was not associated with a decrease in myocardial infarct size	[96]
		Double-blind, placebo-controlled trial to evaluate safety, tolerability, and pharmacokinetics of escalating single intravenous infusion doses of Bendavia (MTP-131)	Patients with heart failure with reduced ejection fraction (ejection fraction, ≤35%) were randomized to either a single 4-h infusion of elamipretide (cohort 1, 0.005; cohort 2, 0.05; and cohort 3, 0.25 mg·kg^−1^·h^−1^) or placebo	A single infusion of elamipretide was safe and well-tolerated. High-dose elamipretide resulted in favorable changes in left ventricular volumes that correlated with peak plasma concentrations, supporting a temporal association and dose-effect relationship	[97]
		Elamipretide in adults with primary mitochondrial myopathy	Participants were randomly assigned (1:1) to 40 mg/day subcutaneous elamipretide for 4 weeks followed by placebo subcutaneous for 4 weeks, separated by a 4-week washout period, or the opposite sequence	Elamipretide was generally well-tolerated and participants who received short-course daily elamipretide for 4 weeks had clinically meaningful improvements in 6 min walk test	[98]
		Randomized, double-blind, placebo-controlled crossover trial followed by an open-label extension to test the effect of elamipretide in Barth syndrome (BTHS)	A group of patients (12 subjects) was randomized to receive 40 mg per day of elamipretide or placebo for 12 weeks, followed by a 4-week washout and then 12 weeks on the opposite arm. Ten subjects continued on the open-label extension (part 2) of 40 mg per day of elamipretide, with 8 subjects reaching 36 weeks	At 36 weeks in part 2, there were significant improvements in 6 min walk test and BTHS Symptom Assessment (BTHS-SA) scale	[99]
Tempol	Pre-clinical study	Animal model of I/R injury	Tempol (30 mg/kg intravenously) prior to and throughout reperfusion	Tempol attenuated renal dysfunction at least partially through reduced renal activity of MPO and level of MDA	[100]
Mito-TEMPO	Pre-clinical study	Animal model of I/R injury	25 μL Mito-tempo was directly injected into each kidney of the mice after reperfusion followed by daily intraperitoneal injection of mito-TEMPO (5 mg/kg) until day 5	Mito-TEMPO restored the renal mtDNA level, mitochondrial mass, and ATP production with consequent reduced inflammation and kidney injury	[101]
XJB-5-131	Pre-clinical study	Animal model of I/R injury	The mice were injected intraperitoneally with XJB-5-131 (10 mg/kg) 30 min prior to ischemia and for 3 consecutive days after surgery	XJB-5-131 attenuated I/R-induced renal injury and inflammation in mice by specifically inhibiting ferroptosis	[102]

## References

[1] Tonelli M., Wiebe N., Knoll G., Bello A., Browne S., Jadhav D., Klarenbach S., Gill J. (2011). Systematic Review: Kidney Transplantation Compared with Dialysis in Clinically Relevant Outcomes. Am. J. Transplant..

[2] Serrano O.K., Vock D.M., Chinnakotla S., Dunn T.B., Kandaswamy R., Pruett T.L., Feldman R., Matas A.J., Finger E.B. (2019). The Relationships Between Cold Ischemia Time, Kidney Transplant Length of Stay, and Transplant-related Costs. Transplantation.

[3] Qureshi F., Rabb H., Kasiske B.L. (2002). Silent acute rejection during prolonged delayed graft function reduces kidney allograft survival. Transplantation.

[4] Yarlagadda S.G., Coca S.G., Formica R.N., Poggio E.D., Parikh C.R. (2009). Association between delayed graft function and allograft and patient survival: A systematic review and meta-analysis. Nephrol. Dial. Transpl..

[5] Perico N., Cattaneo D., Sayegh M.H., Remuzzi G. (2004). Delayed graft function in kidney transplantation. Lancet.

[6] Zaza G., Ferraro P.M., Tessari G., Sandrini S., Scolari M.P., Capelli I., Minetti E., Gesualdo L., Girolomoni G., Gambaro G. (2015). Predictive model for delayed graft function based on easily available pre-renal transplant variables. Intern. Emerg. Med..

[7] Eltzschig H.K., Eckle T. (2011). Ischemia and reperfusion—From mechanism to translation. Nat. Med..

[8] Wu M.Y., Yiang G.T., Liao W.T., Tsai A.P., Cheng Y.L., Cheng P.W., Li C.Y., Li C.J. (2018). Current Mechanistic Concepts in Ischemia and Reperfusion Injury. Cell. Physiol. Biochem..

[9] Mills E.L., Kelly B., O’Neill L.A.J. (2017). Mitochondria are the powerhouses of immunity. Nat. Immunol..

[10] Zhang Q., Raoof M., Chen Y., Sumi Y., Sursal T., Junger W., Brohi K., Itagaki K., Hauser C.J. (2010). Circulating mitochondrial DAMPs cause inflammatory responses to injury. Nature.

[11] Krysko D.V., Agostinis P., Krysko O., Garg A.D., Bachert C., Lambrecht B.N., Vandenabeele P. (2011). Emerging role of damage-associated molecular patterns derived from mitochondria in inflammation. Trends Immunol..

[12] Granata S., Benedetti C., Gambaro G., Zaza G. (2020). Kidney allograft fibrosis: What we learned from latest translational research studies. J. Nephrol..

[13] Masola V., Carraro A., Granata S., Signorini L., Bellin G., Violi P., Lupo A., Tedeschi U., Onisto M., Gambaro G. (2019). In vitro effects of interleukin (IL)-1 beta inhibition on the epithelial-to-mesenchymal transition (EMT) of renal tubular and hepatic stellate cells. J. Transl. Med..

[14] Castellano G., Franzin R., Stasi A., Divella C., Sallustio F., Pontrelli P., Lucarelli G., Battaglia M., Staffieri F., Crovace A. (2018). Complement Activation During Ischemia/Reperfusion Injury Induces Pericyte-to-Myofibroblast Transdifferentiation Regulating Peritubular Capillary Lumen Reduction Through pERK Signaling. Front. Immunol..

[15] Carcy R., Cougnon M., Poet M., Durandy M., Sicard A., Counillon L., Blondeau N., Hauet T., Tauc M., Pisani D.F. (2021). Targeting oxidative stress, a crucial challenge in renal transplantation outcome. Free Radic. Biol. Med..

[16] Granger D.N., Kvietys P.R. (2015). Reperfusion injury and reactive oxygen species: The evolution of a concept. Redox Biol..

[17] Bedard K., Krause K.H. (2007). The NOX family of ROS-generating NADPH oxidases: Physiology and pathophysiology. Physiol. Rev..

[18] Diebold I., Petry A., Hess J., Görlach A. (2010). The NADPH Oxidase Subunit NOX4 Is a New Target Gene of the Hypoxia-inducible Factor-1. Mol. Biol. Cell.

[19] Dana R., Malech H.L., Levy R. (1994). The requirement for phospholipase A2 for activation of the assembled NADPH oxidase in human neutrophils. Biochem. J..

[20] Cui X.-L., Douglas J.G. (1997). Arachidonic acid activates c-jun N-terminal kinase through NADPH oxidase in rabbit proximal tubular epithelial cells. Proc. Natl. Acad. Sci. USA.

[21] Simone S., Rascio F., Castellano G., Divella C., Chieti A., Ditonno P., Battaglia M., Crovace A., Staffieri F., Oortwijn B. (2014). Complement-dependent NADPH oxidase enzyme activation in renal ischemia/reperfusion injury. Free Radic. Biol. Med..

[22] Park H.S., Chun J.N., Jung H.Y., Choi C., Bae Y.S. (2006). Role of NADPH oxidase 4 in lipopolysaccharide-induced proinflammatory responses by human aortic endothelial cells. Cardiovasc. Res..

[23] Bendall J.K., Alp N.J., Warrick N., Cai S., Adlam D., Rockett K., Yokoyama M., Kawashima S., Channon K.M. (2005). Stoichiometric relationships between endothelial tetrahydrobiopterin, endothelial NO synthase (eNOS) activity, and eNOS coupling in vivo: Insights from transgenic mice with endothelial-targeted GTP cyclohydrolase 1 and eNOS overexpression. Circ. Res..

[24] Murphy M.P. (2009). How mitochondria produce reactive oxygen species. Biochem. J..

[25] Chouchani E.T., Pell V.R., Gaude E., Aksentijević D., Sundier S.Y., Robb E.L., Logan A., Nadtochiy S.M., Ord E.N.J., Smith A.C. (2014). Ischaemic accumulation of succinate controls reperfusion injury through mitochondrial ROS. Nature.

[26] Chouchani E.T., Pell V.R., James A.M., Work L.M., Saeb-Parsy K., Frezza C., Krieg T., Murphy M.P. (2016). A Unifying Mechanism for Mitochondrial Superoxide Production during Ischemia-Reperfusion Injury. Cell Metab..

[27] Tang D., Kroemer G. (2020). Ferroptosis. Curr. Biol..

[28] Yagoda N., von Rechenberg M., Zaganjor E., Bauer A.J., Yang W.S., Fridman D.J., Wolpaw A.J., Smukste I., Peltier J.M., Boniface J.J. (2007). RAS-RAF-MEK-dependent oxidative cell death involving voltage-dependent anion channels. Nature.

[29] Dixon S.J., Lemberg K.M., Lamprecht M.R., Skouta R., Zaitsev E.M., Gleason C.E., Patel D.N., Bauer A.J., Cantley A.M., Yang W.S. (2012). Ferroptosis: An iron-dependent form of nonapoptotic cell death. Cell.

[30] Stockwell B.R., Friedmann Angeli J.P., Bayir H., Bush A.I., Conrad M., Dixon S.J., Fulda S., Gascón S., Hatzios S.K., Kagan V.E. (2017). Ferroptosis: A Regulated Cell Death Nexus Linking Metabolism, Redox Biology, and Disease. Cell.

[31] Tang D., Chen X., Kang R., Kroemer G. (2021). Ferroptosis: Molecular mechanisms and health implications. Cell Res..

[32] Gao M., Monian P., Quadri N., Ramasamy R., Jiang X. (2015). Glutaminolysis and Transferrin Regulate Ferroptosis. Mol. Cell.

[33] Gao M., Monian P., Pan Q., Zhang W., Xiang J., Jiang X. (2016). Ferroptosis is an autophagic cell death process. Cell Res..

[34] Bersuker K., Hendricks J.M., Li Z., Magtanong L., Ford B., Tang P.H., Roberts M.A., Tong B., Maimone T.J., Zoncu R. (2019). The CoQ oxidoreductase FSP1 acts parallel to GPX4 to inhibit ferroptosis. Nature.

[35] Hinman A., Holst C.R., Latham J.C., Bruegger J.J., Ulas G., McCusker K.P., Amagata A., Davis D., Hoff K.G., Kahn-Kirby A.H. (2018). Vitamin E hydroquinone is an endogenous regulator of ferroptosis via redox control of 15-lipoxygenase. PLoS ONE.

[36] Miotto G., Rossetto M., Di Paolo M.L., Orian L., Venerando R., Roveri A., Vučković A.M., Bosello Travain V., Zaccarin M., Zennaro L. (2020). Insight into the mechanism of ferroptosis inhibition by ferrostatin-1. Redox Biol..

[37] Friedmann Angeli J.P., Schneider M., Proneth B., Tyurina Y.Y., Tyurin V.A., Hammond V.J., Herbach N., Aichler M., Walch A., Eggenhofer E. (2014). Inactivation of the ferroptosis regulator Gpx4 triggers acute renal failure in mice. Nat. Cell Biol..

[38] Linkermann A., Skouta R., Himmerkus N., Mulay S.R., Dewitz C., De Zen F., Prokai A., Zuchtriegel G., Krombach F., Welz P.S. (2014). Synchronized renal tubular cell death involves ferroptosis. Proc. Natl. Acad. Sci. USA.

[39] Su L., Jiang X., Yang C., Zhang J., Chen B., Li Y., Yao S., Xie Q., Gomez H., Murugan R. (2019). Pannexin 1 mediates ferroptosis that contributes to renal ischemia/reperfusion injury. J. Biol. Chem..

[40] Linden J., Koch-Nolte F., Dahl G. (2019). Purine Release, Metabolism, and Signaling in the Inflammatory Response. Annu. Rev. Immunol..

[41] Sun M., Hao T., Li X., Qu A., Xu L., Hao C., Xu C., Kuang H. (2018). Direct observation of selective autophagy induction in cells and tissues by self-assembled chiral nanodevice. Nat. Commun..

[42] Nalesnik M.A., Gandhi C.R., Starzl T.E. (2017). Augmenter of liver regeneration: A fundamental life protein. Hepatology.

[43] Liao X.H., Zhang L., Liu Q., Sun H., Peng C.M., Guo H. (2010). Augmenter of liver regeneration protects kidneys from ischaemia/reperfusion injury in rats. Nephrol. Dial. Transplant..

[44] Liao X.-H., Chen G.-T., Li Y., Zhang L., Liu Q., Sun H., Guo H. (2012). Augmenter of Liver Regeneration Attenuates Tubular Cell Apoptosis in Acute Kidney Injury in Rats: The Possible Mechanisms. Ren. Fail..

[45] Huang L.L., Liao X.H., Sun H., Jiang X., Liu Q., Zhang L. (2019). Augmenter of liver regeneration protects the kidney from ischaemia-reperfusion injury in ferroptosis. J. Cell. Mol. Med..

[46] Zhu D.J., Liao X.H., Huang W.Q., Sun H., Zhang L., Liu Q. (2020). Augmenter of Liver Regeneration Protects Renal Tubular Epithelial Cells from Ischemia-Reperfusion Injury by Promoting PINK1/Parkin-Mediated Mitophagy. Front. Physiol..

[47] Granata S., Dalla Gassa A., Tomei P., Lupo A., Zaza G. (2015). Mitochondria: A new therapeutic target in chronic kidney disease. Nutr. Metab..

[48] Kubli D.A., Gustafsson Å.B. (2012). Mitochondria and mitophagy: The yin and yang of cell death control. Circ. Res..

[49] Geisler S., Holmström K.M., Skujat D., Fiesel F.C., Rothfuss O.C., Kahle P.J., Springer W. (2010). PINK1/Parkin-mediated mitophagy is dependent on VDAC1 and p62/SQSTM1. Nat. Cell Biol..

[50] Kane L.A., Lazarou M., Fogel A.I., Li Y., Yamano K., Sarraf S.A., Banerjee S., Youle R.J. (2014). PINK1 phosphorylates ubiquitin to activate Parkin E3 ubiquitin ligase activity. J. Cell Biol..

[51] Deas E., Plun-Favreau H., Gandhi S., Desmond H., Kjaer S., Loh S.H., Renton A.E., Harvey R.J., Whitworth A.J., Martins L.M. (2011). PINK1 cleavage at position A103 by the mitochondrial protease PARL. Hum. Mol. Genet..

[52] Youle R.J., Narendra D.P. (2011). Mechanisms of mitophagy. Nat. Rev. Mol. Cell Biol..

[53] Ashrafi G., Schwarz T.L. (2013). The pathways of mitophagy for quality control and clearance of mitochondria. Cell Death Differ..

[54] Esteban-Martínez L., Boya P. (2018). BNIP3L/NIX-dependent mitophagy regulates cell differentiation via metabolic reprogramming. Autophagy.

[55] Scott I., Youle R.J. (2010). Mitochondrial fission and fusion. Essays Biochem..

[56] Twig G., Elorza A., Molina A.J.A., Mohamed H., Wikstrom J.D., Walzer G., Stiles L., Haigh S.E., Katz S., Las G. (2008). Fission and selective fusion govern mitochondrial segregation and elimination by autophagy. EMBO J..

[57] Chen M., Chen Z., Wang Y., Tan Z., Zhu C., Li Y., Han Z., Chen L., Gao R., Liu L. (2016). Mitophagy receptor FUNDC1 regulates mitochondrial dynamics and mitophagy. Autophagy.

[58] Ishihara M., Urushido M., Hamada K., Matsumoto T., Shimamura Y., Ogata K., Inoue K., Taniguchi Y., Horino T., Fujieda M. (2013). Sestrin-2 and BNIP3 regulate autophagy and mitophagy in renal tubular cells in acute kidney injury. Am. J. Physiol. Physiol..

[59] Tang C., Han H., Yan M., Zhu S., Liu J., Liu Z., He L., Tan J., Liu Y., Liu H. (2018). PINK1-PRKN/PARK2 pathway of mitophagy is activated to protect against renal ischemia-reperfusion injury. Autophagy.

[60] Tang C., Han H., Liu Z., Liu Y., Yin L., Cai J., He L., Liu Y., Chen G., Zhang Z. (2019). Activation of BNIP3-mediated mitophagy protects against renal ischemia-reperfusion injury. Cell Death Dis..

[61] Fu Z.J., Wang Z.Y., Xu L., Chen X.H., Li X.X., Liao W.T., Ma H.K., Jiang M.D., Xu T.T., Xu J. (2020). HIF-1α-BNIP3-mediated mitophagy in tubular cells protects against renal ischemia/reperfusion injury. Redox Biol..

[62] Li N., Wang H., Jiang C., Zhang M. (2018). Renal ischemia/reperfusion-induced mitophagy protects against renal dysfunction via Drp1-dependent-pathway. Exp. Cell Res..

[63] Feng J., Li H., Zhang Y., Wang Q., Zhao S., Meng P., Li J. (2018). Mammalian STE20-Like Kinase 1 Deletion Alleviates Renal Ischaemia-Reperfusion Injury via Modulating Mitophagy and the AMPK-YAP Signalling Pathway. Cell Physiol. Biochem..

[64] Livingston M.J., Wang J., Zhou J., Wu G., Ganley I.G., Hill J.A., Yin X.-M., Dong Z. (2019). Clearance of damaged mitochondria via mitophagy is important to the protective effect of ischemic preconditioning in kidneys. Autophagy.

[65] Kharbanda R.K., Nielsen T.T., Redington A.N. (2009). Translation of remote ischaemic preconditioning into clinical practice. Lancet.

[66] Tong F., Zhou X. (2017). The Nrf2/HO-1 Pathway Mediates the Antagonist Effect of L-Arginine on Renal Ischemia/Reperfusion Injury in Rats. Kidney Blood Press. Res..

[67] Itoh K., Wakabayashi N., Katoh Y., Ishii T., Igarashi K., Engel J.D., Yamamoto M. (1999). Keap1 represses nuclear activation of antioxidant responsive elements by Nrf2 through binding to the amino-terminal Neh2 domain. Genes Dev..

[68] Itoh K., Chiba T., Takahashi S., Ishii T., Igarashi K., Katoh Y., Oyake T., Hayashi N., Satoh K., Hatayama I. (1997). An Nrf2/small Maf heterodimer mediates the induction of phase II detoxifying enzyme genes through antioxidant response elements. Biochem. Biophys. Res. Commun..

[69] Nezu M., Souma T., Yu L., Suzuki T., Saigusa D., Ito S., Suzuki N., Yamamoto M. (2017). Transcription factor Nrf2 hyperactivation in early-phase renal ischemia-reperfusion injury prevents tubular damage progression. Kidney Int..

[70] Agyeman A.S., Chaerkady R., Shaw P.G., Davidson N.E., Visvanathan K., Pandey A., Kensler T.W. (2012). Transcriptomic and proteomic profiling of KEAP1 disrupted and sulforaphane-treated human breast epithelial cells reveals common expression profiles. Breast Cancer Res. Treat..

[71] Harada N., Kanayama M., Maruyama A., Yoshida A., Tazumi K., Hosoya T., Mimura J., Toki T., Maher J.M., Yamamoto M. (2011). Nrf2 regulates ferroportin 1-mediated iron efflux and counteracts lipopolysaccharide-induced ferroportin 1 mRNA suppression in macrophages. Arch. Biochem. Biophys..

[72] Jiang G.P., Liao Y.J., Huang L.L., Zeng X.J., Liao X.H. (2021). Effects and molecular mechanism of pachymic acid on ferroptosis in renal ischemia reperfusion injury. Mol. Med. Rep..

[73] Yang H., Magilnick N., Lee C., Kalmaz D., Ou X., Chan J.Y., Lu S.C. (2005). Nrf1 and Nrf2 regulate rat glutamate-cysteine ligase catalytic subunit transcription indirectly via NF-kappaB and AP-1. Mol. Cell Biol..

[74] Liu M., Grigoryev D.N., Crow M.T., Haas M., Yamamoto M., Reddy S.P., Rabb H. (2009). Transcription factor Nrf2 is protective during ischemic and nephrotoxic acute kidney injury in mice. Kidney Int..

[75] Siegel D., Gustafson D.L., Dehn D.L., Han J.Y., Boonchoong P., Berliner L.J., Ross D. (2004). NAD(P)H:quinone oxidoreductase 1: Role as a superoxide scavenger. Mol. Pharmacol..

[76] Gang G.T., Hwang J.H., Kim Y.H., Noh J.R., Kim K.S., Jeong J.Y., Choi D.E., Lee K.W., Jung J.Y., Shong M. (2014). Protection of NAD(P)H:quinone oxidoreductase 1 against renal ischemia/reperfusion injury in mice. Free Radic. Biol. Med..

[77] Corsello T., Komaravelli N., Casola A. (2018). Role of Hydrogen Sulfide in NRF2- and Sirtuin-Dependent Maintenance of Cellular Redox Balance. Antioxidants.

[78] Yang G., Zhao K., Ju Y., Mani S., Cao Q., Puukila S., Khaper N., Wu L., Wang R. (2013). Hydrogen sulfide protects against cellular senescence via S-sulfhydration of Keap1 and activation of Nrf2. Antioxid. Redox Signal..

[79] Tripatara P., Patel N.S., Collino M., Gallicchio M., Kieswich J., Castiglia S., Benetti E., Stewart K.N., Brown P.A., Yaqoob M.M. (2008). Generation of endogenous hydrogen sulfide by cystathionine gamma-lyase limits renal ischemia/reperfusion injury and dysfunction. Lab. Investig..

[80] Tripatara P., Patel N.S., Brancaleone V., Renshaw D., Rocha J., Sepodes B., Mota-Filipe H., Perretti M., Thiemermann C. (2009). Characterisation of cystathionine gamma-lyase/hydrogen sulphide pathway in ischaemia/reperfusion injury of the mouse kidney: An in vivo study. Eur. J. Pharmacol..

[81] Hourihan J.M., Kenna J.G., Hayes J.D. (2013). The gasotransmitter hydrogen sulfide induces nrf2-target genes by inactivating the keap1 ubiquitin ligase substrate adaptor through formation of a disulfide bond between cys-226 and cys-613. Antioxid. Redox Signal..

[82] Bos E.M., Leuvenink H.G., Snijder P.M., Kloosterhuis N.J., Hillebrands J.L., Leemans J.C., Florquin S., van Goor H. (2009). Hydrogen sulfide-induced hypometabolism prevents renal ischemia/reperfusion injury. J. Am. Soc. Nephrol..

[83] Han S.J., Kim J.I., Park J.W., Park K.M. (2015). Hydrogen sulfide accelerates the recovery of kidney tubules after renal ischemia/reperfusion injury. Nephrol. Dial. Transpl..

[84] Zhao H., Qiu Y., Wu Y., Sun H., Gao S. (2021). Protective Effects of GYY4137 on Renal Ischaemia/Reperfusion Injury through Nrf2-Mediated Antioxidant Defence. Kidney Blood Press. Res..

[85] Blackstone E., Morrison M., Roth M.B. (2005). H_2_S induces a suspended animation-like state in mice. Science.

[86] Blackstone E., Roth M.B. (2007). Suspended animation-like state protects mice from lethal hypoxia. Shock.

[87] Kezic A., Spasojevic I., Lezaic V., Bajcetic M. (2016). Mitochondria-Targeted Antioxidants: Future Perspectives in Kidney Ischemia Reperfusion Injury. Oxid. Med. Cell. Longev..

[88] Dare A.J., Bolton E.A., Pettigrew G.J., Bradley J.A., Saeb-Parsy K., Murphy M.P. (2015). Protection against renal ischemia-reperfusion injury in vivo by the mitochondria targeted antioxidant MitoQ. Redox Biol..

[89] Liu X., Murphy M.P., Xing W., Wu H., Zhang R., Sun H. (2018). Mitochondria-targeted antioxidant MitoQ reduced renal damage caused by ischemia-reperfusion injury in rodent kidneys: Longitudinal observations of T2 -weighted imaging and dynamic contrast-enhanced MRI. Magn. Reason. Med..

[90] Rossman M.J., Santos-Parker J.R., Steward C.A.C., Bispham N.Z., Cuevas L.M., Rosenberg H.L., Woodward K.A., Chonchol M., Gioscia-Ryan R.A., Murphy M.P. (2018). Chronic Supplementation with a Mitochondrial Antioxidant (MitoQ) Improves Vascular Function in Healthy Older Adults. Hypertension.

[91] Snow B.J., Rolfe F.L., Lockhart M.M., Frampton C.M., O’Sullivan J.D., Fung V., Smith R.A., Murphy M.P., Taylor K.M., Protect Study Group (2010). A double-blind, placebo-controlled study to assess the mitochondria-targeted antioxidant MitoQ as a disease-modifying therapy in Parkinson’s disease. Mov. Disord..

[92] Gane E.J., Weilert F., Orr D.W., Keogh G.F., Gibson M., Lockhart M.M., Frampton C.M., Taylor K.M., Smith R.A., Murphy M.P. (2010). The mitochondria-targeted anti-oxidant mitoquinone decreases liver damage in a phase II study of hepatitis C patients. Liver Int..

[93] Szeto H.H., Liu S., Soong Y., Seshan S.V., Cohen-Gould L., Manichev V., Feldman L.C., Gustafsson T. (2017). Mitochondria Protection after Acute Ischemia Prevents Prolonged Upregulation of IL-1β and IL-18 and Arrests CKD. J. Am. Soc. Nephrol..

[94] Wyss J.C., Kumar R., Mikulic J., Schneider M., Mary J.L., Aebi J.D., Juillerat-Jeanneret L., Golshayan D. (2019). Differential Effects of the Mitochondria-Active Tetrapeptide SS-31 (D-Arg-dimethylTyr-Lys-Phe-NH2) and Its Peptidase-Targeted Prodrugs in Experimental Acute Kidney Injury. Front. Pharmacol..

[95] Saad A., Herrmann S.M.S., Eirin A., Ferguson C.M., Glockner J.F., Bjarnason H., McKusick M.A., Misra S., Lerman L.O., Textor S.C. (2017). Phase 2a Clinical Trial of Mitochondrial Protection (Elamipretide) During Stent Revascularization in Patients with Atherosclerotic Renal Artery Stenosis. Circ. Cardiovasc. Interv..

[96] Chakrabarti A.K., Feeney K., Abueg C., Brown D.A., Czyz E., Tendera M., Janosi A., Giugliano R.P., Kloner R.A., Weaver W.D. (2013). Rationale and design of the EMBRACE STEMI study: A phase 2a, randomized, double-blind, placebo-controlled trial to evaluate the safety, tolerability and efficacy of intravenous Bendavia on reperfusion injury in patients treated with standard therapy including primary percutaneous coronary intervention and stenting for ST-segment elevation myocardial infarction. Am. Heart J..

[97] Daubert M.A., Yow E., Dunn G., Marchev S., Barnhart H., Douglas P.S., O’Connor C., Goldstein S., Udelson J.E., Sabbah H.N. (2017). Novel Mitochondria-Targeting Peptide in Heart Failure Treatment: A Randomized, Placebo-Controlled Trial of Elamipretide. Circ. Heart Fail..

[98] Karaa A., Haas R., Goldstein A., Vockley J., Cohen B.H. (2020). A randomized crossover trial of elamipretide in adults with primary mitochondrial myopathy. J. Cachexia Sarcopenia Muscle.

[99] Reid Thompson W., Hornby B., Manuel R., Bradley E., Laux J., Carr J., Vernon H.J. (2021). A phase 2/3 randomized clinical trial followed by an open-label extension to evaluate the effectiveness of elamipretide in Barth syndrome, a genetic disorder of mitochondrial cardiolipin metabolism. Genet. Med..

[100] Chatterjee P.K., Cuzzocrea S., Brown P.A., Zacharowski K., Stewart K.N., Mota-Filipe H., Thiemermann C. (2000). Tempol, a membrane-permeable radical scavenger, reduces oxidant stress-mediated renal dysfunction and injury in the rat. Kidney Int..

[101] Zhao M., Wang Y., Li L., Liu S., Wang C., Yuan Y., Yang G., Chen Y., Cheng J., Lu Y. (2021). Mitochondrial ROS promote mitochondrial dysfunction and inflammation in ischemic acute kidney injury by disrupting TFAM-mediated mtDNA maintenance. Theranostics.

[102] Zhao Z., Wu J., Xu H., Zhou C., Han B., Zhu H., Hu Z., Ma Z., Ming Z., Yao Y. (2020). XJB-5-131 inhibited ferroptosis in tubular epithelial cells after ischemia-reperfusion injury. Cell Death Dis..

[103] Kelso G.F., Porteous C.M., Coulter C.V., Hughes G., Porteous W.K., Ledgerwood E.C., Smith R.A., Murphy M.P. (2001). Selective targeting of a redox-active ubiquinone to mitochondria within cells: Antioxidant and antiapoptotic properties. J. Biol. Chem..

[104] Smith R.A., Murphy M.P. (2010). Animal and human studies with the mitochondria-targeted antioxidant MitoQ. Ann. N. Y. Acad. Sci..

[105] Jelinek A., Heyder L., Daude M., Plessner M., Krippner S., Grosse R., Diederich W.E., Culmsee C. (2018). Mitochondrial rescue prevents glutathione peroxidase-dependent ferroptosis. Free Radic. Biol. Med..

[106] Birk A.V., Liu S., Soong Y., Mills W., Singh P., Warren J.D., Seshan S.V., Pardee J.D., Szeto H.H. (2013). The mitochondrial-targeted compound SS-31 re-energizes ischemic mitochondria by interacting with cardiolipin. J. Am. Soc. Nephrol..

[107] Zhao K., Zhao G.M., Wu D., Soong Y., Birk A.V., Schiller P.W., Szeto H.H. (2004). Cell-permeable peptide antioxidants targeted to inner mitochondrial membrane inhibit mitochondrial swelling, oxidative cell death, and reperfusion injury. J. Biol. Chem..

[108] Laight D.W., Andrews T.J., Haj-Yehia A.I., Carrier M.J., Anggård E.E. (1997). Microassay of superoxide anion scavenging activity in vitro. Environ. Toxicol. Pharmacol..

[109] Krishna M.C., Russo A., Mitchell J.B., Goldstein S., Dafni H., Samuni A. (1996). Do nitroxide antioxidants act as scavengers of $O_{2}^{\bar{\cdot }}$
or as SOD mimics?. J. Biol. Chem..

[110] Trnka J., Blaikie F.H., Smith R.A., Murphy M.P. (2008). A mitochondria-targeted nitroxide is reduced to its hydroxylamine by ubiquinol in mitochondria. Free Radic. Biol. Med..

[111] Apostolova N., Victor V.M. (2015). Molecular strategies for targeting antioxidants to mitochondria: Therapeutic implications. Antioxid. Redox Signal..

[112] Bao N., Dai D. (2020). Dexmedetomidine Protects against Ischemia and Reperfusion-Induced Kidney Injury in Rats. Mediat. Inflamm..

[113] Cakir M., Polat A., Tekin S., Vardi N., Taslidere E., Rumeysa Duran Z., Tanbek K. (2015). The effect of dexmedetomidine against oxidative and tubular damage induced by renal ischemia reperfusion in rats. Ren. Fail..

[114] Kocoglu H., Ozturk H., Ozturk H., Yilmaz F., Gulcu N. (2009). Effect of dexmedetomidine on ischemia-reperfusion injury in rat kidney: A histopathologic study. Ren. Fail..

[115] Gu J., Sun P., Zhao H., Watts H.R., Sanders R.D., Terrando N., Xia P., Maze M., Ma D. (2011). Dexmedetomidine provides renoprotection against ischemia-reperfusion injury in mice. Crit. Care.

[116] Zhao X., Zhang E., Ren X., Bai X., Wang D., Bai L., Luo D., Guo Z., Wang Q., Yang J. (2020). Edaravone alleviates cell apoptosis and mitochondrial injury in ischemia-reperfusion-induced kidney injury via the JAK/STAT pathway. Biol. Res..

[117] Doi K., Suzuki Y., Nakao A., Fujita T., Noiri E. (2004). Radical scavenger edaravone developed for clinical use ameliorates ischemia/reperfusion injury in rat kidney. Kidney Int..

[118] Zilka O., Shah R., Li B., Friedmann Angeli J.P., Griesser M., Conrad M., Pratt D.A. (2017). On the Mechanism of Cytoprotection by Ferrostatin-1 and Liproxstatin-1 and the Role of Lipid Peroxidation in Ferroptotic Cell Death. ACS Cent. Sci..

